# New Insights Into Functions of IQ67-Domain Proteins

**DOI:** 10.3389/fpls.2020.614851

**Published:** 2021-02-18

**Authors:** Chunyue Guo, Jun Zhou, Dengwen Li

**Affiliations:** ^1^State Key Laboratory of Medicinal Chemical Biology, College of Life Sciences, Nankai University, Tianjin, China; ^2^Institute of Biomedical Sciences, Shandong Provincial Key Laboratory of Animal Resistance Biology, Collaborative Innovation Center of Cell Biology in Universities of Shandong, College of Life Sciences, Shandong Normal University, Jinan, China

**Keywords:** IQD, scaffold proteins, cortical microtubules, microtubule dynamics, organ shape

## Abstract

IQ67-domain (IQD) proteins, first identified in *Arabidopsis* and rice, are plant-specific calmodulin-binding proteins containing highly conserved motifs. They play a critical role in plant defenses, organ development and shape, and drought tolerance. Driven by comprehensive genome identification and analysis efforts, IQDs have now been characterized in several species and have been shown to act as microtubule-associated proteins, participating in microtubule-related signaling pathways. However, the precise molecular mechanisms underpinning their biological functions remain incompletely understood. Here we review current knowledge on how IQD family members are thought to regulate plant growth and development by affecting microtubule dynamics or participating in microtubule-related signaling pathways in different plant species and propose some new insights.

## Introduction

IQ67-domain (IQD) proteins, originally identified in *Arabidopsis thaliana* and rice (Abel et al., 2005), are a class of calmodulin-binding proteins unique to plants (Levy et al., 2005). They are common in a wide variety of land plants from moss to vascular plants, and they play a critical role in basic host defenses (Abel et al., 2005; Levy et al., 2005), cell shaping (Huang et al., 2013; Liu et al., 2020), and drought resistance (Ma et al., 2014; Wu et al., 2016; Yuan et al., 2019). The proteins locate to various compartments including the nucleus, cytoplasm, plasma membrane, and microtubules in *Arabidopsis* (Burstenbinder et al., 2017b), but their subcellular localization patterns vary (Tables 1, 2).

**TABLE 1:** Subcellular localization and subfamilies members of IQD genes in the plant species.

	*Arabidopsis thaliana*	*Oryza sativa*	*Solanum lycopersicum*	*Brachypodium distachyon*	*Populus trichocarpa*	*Glycine max*	*Phyllostachys edulis*		*Cucumis sativus*	*Brassica rapa*	*Vitis vinifera*
Subcellular localization	Nucleus Membrane Microtubules	Nucleus cytoplasm Microtubules		cell nucleus chloroplasts, mitochondria	65% nucleus, chloroplasts, mitochondria, plasma membrane, endoplasmic reticulum (ER)	Nucleus endoplasmic reticulum, chloroplast				plasma membrane	nucleus and membrane
subfamilies	I∼IV	I∼IV	I∼IV	Main group I (Subgroup A, B, and C) and II	I∼IV	I∼IV	I-IV	I-IV	I-IV	I-III	eight subgroups
References	Burstenbinder et al., 2017b	Yang et al., 2020	Feng et al., 2014	Filiz et al., 2013	Ma et al., 2014	Feng et al., 2014	Wu et al., 2016	Cai et al., 2016	Ge et al., 2019	Yuan et al., 2019	Liu et al., 2020

**TABLE 2:** List of IQD genes identified in virous plant species.

Species	Name	Mem	Chr^a^	Length (aa)	pI	Orthologous relationships	References
*Arabidopsis thaliana*	AtIQD	33	5/5	103-794	8.5-11.3 (10.3)	OsIQD	Abel et al., 2005
*Oryza sativa*	OsIQD	29	Mainly 1,5,3	303-893	8.3-11.5 (10.04)	AtIQD	Abel et al., 2005
*Solanum lycopersicum*	SUN	34	12/12	128-862		AtIQD	Huang et al., 2013
*Brachypodium distachyon*	BdIQD	23	12/12	340-585	6.44-11.52 22↑ ≥ 7	OsIQD;OsIQD	Filiz et al., 2013
*Populus trichocarpa*	PtIQD	40	18/19	135-819(464)	10.3 ± 0.6	AtIQD	Ma et al., 2014
*Glycine max*	GmIQD	67	20/20	141-904	5.4-11.1	SUN	Feng et al., 2014
*Phyllostachys edulis*	PeIQD	29		190-940	5.02-11.12	OsIQD	Wu et al., 2016
*Zea mays*	ZmIQD	26	8/10	326-582	9.78-11.4	OsIQD;BdIQD	Cai et al., 2016
*Cucumis sativus*	CsIQD	28	7/7	261-1558			Ge et al., 2019
*Brassica rapa*	BrIQD	35	9/10	290-744	5.42-11.46 (10.05)	AtIQD(13 pairs)	Yuan et al., 2019
*Vitis vinifera*	VvIQD	49	19/19	137-1558 (732.76)	4.72-11.02	AtIQD	Liu et al., 2020

*^*a*^Chromosomal locations of various species *IQD* genes.*

IQD proteins have a central region of 67 conserved amino acids, the eponymous IQ67 domain, which is responsible for recruiting calmodulin, which acts as a Ca^2+^ sensor (Abel et al., 2013). There are two types of IQ67 domain: (1) the Ca^2+^-independent IQ motif, the IQ motif (IQxxxRGxxxR or I/L/VQxxxRxxxxR/K); and (2) the Ca^2+^-dependent IQ motifs, the 1-5-10 and 1-8-14 motifs. The IQ motif includes 1-3 IQ xxxRGxxxR/[ILV]QxxxRxxxx[RK], the 1-5-10 motif contains 1-4 [FILVW]x3[FILV]x4[FILVW], while the 1-8-14 motif contains 1-4 [FILVW]x6[FAILVW]x5[FILVW](Abel et al., 2005; Wu et al., 2011). The IQD protein family has now been comprehensively annotated in several plants (Table 2). Even though their functions differ in some plants studied, for example, SUN/IQD regulates cell division to elongate tomatoes (Wu et al., 2011); IQD1 acts as a defense against herbivores such as aphids in *Arabidopsis* (Abel et al., 2005; Levy et al., 2005); while ZmIQDs and PtIQDs respond to drought stress (Ma et al., 2014; Cai et al., 2016), the underlying molecular basis or the function of other undefined IQDs in different plants may share same mechanisms, but this has not been confirmed.

## IQD Proteins, the Scaffold Proteins Associated Microtubules

Scaffolding proteins interact or bind with several proteins to form an anchoring complex in specific intracellular niches such as the cell membrane, cytoplasmic matrix, or nucleus, and they play an important role in signal transduction. As scaffolding proteins, IQDs play an important role in plant growth and development (Abel et al., 2013; Burstenbinder et al., 2013, 2017a) and link Ca^2+^ signals with some organelles (Burstenbinder et al., 2017b). Yeast two-hybrid and pulldown experiments have verified that *Arabidopsis* IQD1 and IQD20 interact with CaM/CaML both *in vivo* and *in vitro*.

Kinesin light chain is generally located at the end of kinesin and participates in cargo transport (Saez et al., 2020). Therefore, IQD may co-localize with microtubules in addition to its classic nuclear localization, a finding subsequently confirmed using high-resolution fluorescence microscopy. IQD1 interacted with KLCR1 and CaM, thereby linking kinesin to Ca^2+^ second messenger signaling (Steinhorst and Kudla, 2013; Bi et al., 2018). Other IQD family proteins may also mediate different kinesin-dependent cargo transport signaling pathways such as protein sorting or cell wall formation (Kong et al., 2015), and these proteins and interactions require further study.

## Abnormal Shoot 6 and Cortical Microtubules

Microtubules in plant cells are non-centrosome microtubule organized (Paradez et al., 2006; Wasteneys and Ambrose, 2009). Cortical microtubules (CMTs) in the interphase, preprophase band (PPB), spindle and the membrane forming body (phragmoplast) in the mitosis cell form the plant-specific microtubule arrays (Hamada, 2014). Cortical microtubules (CMTs) determine the shape of plant cells (Wasteneys and Ambrose, 2009). Usually MT-associated proteins (MAPs) interact with cortical microtubules to regulate cell shape, such as Augmin complex, Katanin, SPR2, MOR1 and so on (Chen et al., 2016). However, the dynamic regulation of cortical microtubule arrays is complex, which need further studied.

**FIGURE 1:**
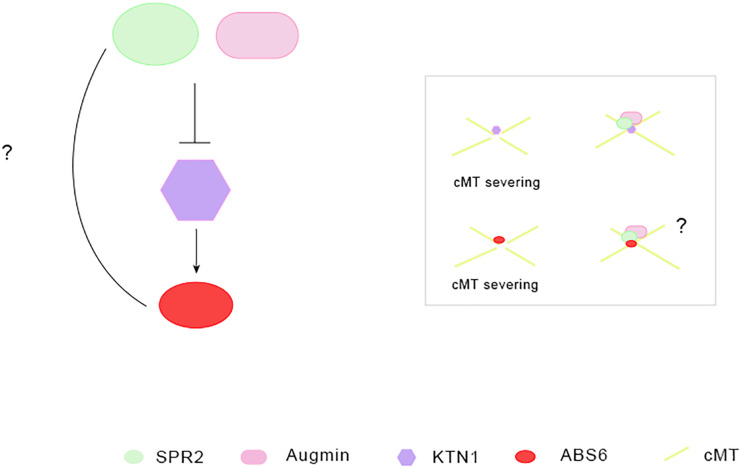
The role of microtubule-associated proteins in cortical microtubule severing and ordering. SPR2, Augmin localized in the cMT crossover sites can prevents KTN1-mediated cMTs severing and ordering (Wightman et al., 2013; Wang et al., 2018; Tian and Kong, 2019). KATANIN 1 (KTN1): p60 catalytic subunit of MT cleavage enzyme katanin, promotes cortical microtubule severing and ordering. It is the positive regulator of ABS6 in cortical microtubule severing and ordering (Li et al., 2020). ABS6, a plant-specific IQD protein and MAP, promotes cortical microtubule severing and ordering (Li et al., 2020).

Li et al., 2020 first identified two previously unknown plant-specific positive regulators of cMT severing and ordering, ABNORMAL SHOOT 6 (ABS6) and SHADE AVOIDANCE 4 (SAV4). ABS6 binds to MT through its C-terminal and it is a kind of plant-specific IQD protein (Li et al., 2020). KATANIN 1 (KTN1), the p60 catalytic subunit of the classical MT-severing enzyme katanin, positively regulate ABS6-mediated cMT severing (Li et al., 2020). Augmin complexes and SPR2 located to the cMT crossover sites suppress KTN1-mediated cMT severing (Wightman et al., 2013; Wang et al., 2018; Tian and Kong, 2019). However, it is not known whether SPR2 inhibit the microtubule cleavage function of ABS6 directly, and whether SPR2 interacts with ABS6 (Figure 1), similar to the direct physical interaction between ABS6, SAV4, and KTN1. Additionally, only half of the C-end of ABS6 is combined with MT, which is also an interesting issue to be explored. If, as Li et al. (2020) guess, other proteins are required to adjust the conformation of ABS6 to make the full-length ABS6 interact with KTN1 and SAV4. Which proteins can regulate its conformation, has not been studied so far.

## The Role of IQD on Organ Shape

### *Arabidopsis* IQD5 and Pavement Cell Shape

Pavement cell are tightly packed in plant epidermis, with many lobes (Cosgrove, 2018; Cosgrove and Anderson, 2020). The lobes formation would be related to the dynamics of the cytoskeleton (Panteris and Galatis, 2005; Cosgrove and Anderson, 2020).

Disordered cortical microtubules usually correlate with wider pavement cell indentations and reduced lobe length. Due to the abnormal expression of IQD5, IQD11, IQD14, IQD16, and IQD25 in *Arabidopsis*, cortical microtubules become disordered in pavement cells to affect their shape, indicating that IQD proteins may regulate anisotropic growth and shape formation by regulating the order of cortical microtubules (Burstenbinder et al., 2017b; Liang et al., 2018; Mitra et al., 2019). Different IQDs affect microtubule organization in different ways to produce unique phenotypes (Liang et al., 2018). Due to the limitations of intracellular Ca^2+^ imaging and the functional redundancy of the IQD family, the specific regulatory mechanisms are still unclear (Mitra et al., 2019). It is complex.

IQD5 is highly expressed in the vegetative organs of plants and combines evenly across cortical microtubules (Liang et al., 2018). In iqd5-1 mutants, microtubule stability decreases, thereby disordering microtubules in cotyledon cells and decreasing the interdigitation of pavement cells. Therefore, IQD5 stabilizes microtubules by decreasing their dynamics. In *Arabidopsis* M2 seedlings, pavement cells in *IQD5* mutants (bQ18E, iqd5-1, and iqd5-2) lack interdigitating lobes compared to wild-type Col-0, with cells becoming smaller and rounder. In three-day-old cotyledons, leaf length is reduced and the neck width is increased in mutants. IQD5 therefore plays an essential role in regulating *Arabidopsis* leaf morphogenesis (Liang et al., 2018). However, the mechanisms of IQD5 affecting leaf morphogenesis remain to be explored.

Furthermore, Ca^2+^ signaling plays a key role for the pavement cell morphology and IQD5’s recruitment to cortical microtubules (Mitra et al., 2019). The IQD-KLCR module stabilizes cortical microtubules laterally, especially at the microtubule-plasma membrane interface (Mitra et al., 2019). Unlike IQD5, which inhibits microtubule dynamics to stabilize microtubules, microtubule-associated proteins exist in *Arabidopsis* that affect microtubule organization by promoting their growth, contraction, and catastrophe frequency, thereby enhancing microtubule dynamics and ensuring normal sorting (Liang et al., 2018) [e.g., MOR1 in the *Arabidopsis* MAP215 family (Twell et al., 2002)]. This coordinated regulation of microtubule dynamics by different proteins enables microtubule cytoskeletal organization, nucleation, and severing. Intracellular signals are thereby transmitted in an ordered manner to control normal plant development (Liang et al., 2018).

### OsIQD14 and the Shape of Seed in Rice

Rice is an important crop that has been subject to extensive efforts to increase grain size and yields. Rice OsIQD14 (Yang et al., 2020), an IQD family protein, is highly expressed in rice seed hull cells, regulating microtubule cytoskeletal dynamics to control rice grain size. In addition to localizing to the nucleus and cytoplasm, OsIQD14 also distributes along microtubules. When OsIQD14 is depleted, grains become wider and shorter and crop yields increase; when OsIQD14 is overexpressed, grains become longer and narrower without an effect on overall yield. OsIQD14 interacts with MAPs to cause catastrophic events such as expansion and contraction, thereby reducing microtubule dynamics to form narrower cells. The IQD C-terminus binds to microtubules, and the IQ67 region at the N-terminus interacts with CAM; both proteins are located on microtubules.

However, the specific molecular mechanism of IQD affecting the shape of rice seeds, such as how to respond to Ca2 + signals to affect the interaction between IQD and CaM remains to be explored. Breeding has traditionally been manipulated by altering intracellular signal transduction through GW5 and GW5L (Duan et al., 2017; Liu et al., 2017). GW5 is an IQD protein located in the plasma membrane and is involved in brassinosteroid signaling. And It is similar to OsIQD14 about its regulation of seed shape (Duan et al., 2017; Liu et al., 2017; Yang et al., 2020).

OsIQD14 controls cytoskeletal dynamics and cell morphology in rice by integrating auxin and calcium signaling pathways to increase rice yield. Regarding its specific mechanism, many hypotheses have been proposed, including the interaction among OsIQD14, SPR2 and CaM proteins is regulated by auxin/blue light and Ca2 + signal (Yang et al., 2020). Moreover, it is unclear whether there are other microtubule-related proteins such as katanin, MOR1, and Augmin involved with the process, and how they regulate microtubule dynamics and respond to environment signals.

### IQD/SUN in Tomato

The tomato plant is a useful model for studying fleshy fruit development. Since the *Solanum lycopersicum* genome is small and highly conserved, it serves as a reference for other species in the Solanaceae family such as peppers, eggplants, and potatoes (Wu et al., 2016). Due to improvements in living standards and cultural changes, new fruits and vegetables such as square watermelons, large green peppers, and long tomatoes are now of commercial interest. Therefore, the study of genes that regulate the shape of edible plant organs is of increasing interest. The microtubule-binding proteins IQD/SUN, OFP (ovate family protein), and TRM (TON1 recruiting motif proteins) can interact with each other to form complexes and combine with microtubules to regulate microtubule-related pathways and ultimately affect tomato fruit shape (van der Knaap et al., 2014; Lazzaro et al., 2018; Wu et al., 2018). SUN, OVATE, and TRM are all implicated in tomato shaping (Xiao et al., 2008, 2009; Wu et al., 2016). IQD is a microtubule-binding protein, and TRM is also located in microtubules (Lee et al., 2006; Drevensek et al., 2012). Ovate is the archetypal OFP, and while OFPs are mostly nuclear, the OFP-TRM complex migrates through the cell to bind to microtubules (Lazzaro et al., 2018; Snouffer et al., 2020). IQD/SUN and TRM elongate tomatoes, while ovate (OFP) inhibits elongation. IQD12 controls fruit elongation via alterations to cell division patterning, while TRM1-5-like genes promote the elongation of fruits, grains, leaves, and tubers, with OFP1 having the opposite effect (Wu et al., 2011, 2018; Lazzaro et al., 2018).

IQD locates to microtubules and regulates microtubule dynamics by interacting with KLCR, CMU (Cellulose-Microtubule Uncoupling), and other related proteins. AtIQD5 may mediate the coupling of cellulose synthase movement to orbital microtubules, and cortical microtubules act as the template to transport CSCs to the plasma membrane. The slightest deviation to the trajectory of anchoring to the cell wall will directly affect the cell wall positioning of CSCs, consequently affecting the directional deposition of cellulose in the cell wall and the direction of cell expansion (Endler and Persson, 2011); ultimately, this will change the cell shape and the organ. AtOPF4 directly affects cell wall formation by interacting with KNAT7 (Li et al., 2011). Furthermore, cell division is affected by Pok1, which is mainly regulated by TRM, as well as the interaction between Pok1 and ROPs (Rho-like GTPases). These proteins also locate to the PPB, spindle, and phragmoplast. OFP and TRM regulate cell division during ovary development (Wu et al., 2018). Similarly, AtIQD5 also localizes to the PPB, spindle, and cortical microtubules in roots. Moreover, OPFs, TRMs, and TTP complexes are involved in cell plate positioning during cell division, which in turn affects organ shape.

## Conclusion and Perspectives

In addition to affecting the shape of the cells and organs of some plants, IQDs can also enhance drought resistance of some plants including cabbage, corn, moso bamboo, and poplar (Ma et al., 2014; Cai et al., 2016; Wu et al., 2016; Yuan et al., 2019). The 26 ZmIQD genes in maize are regulated by drought stress. BrIQD5 is a potential target gene to improve the drought tolerance of cabbage, and four drought-related proteins have been found to interact with BrIQD5. However, this work remains in its infancy, and the IQD-related molecular pathways underpinning drought resistance need further study.

For the important role of IQD in plants, we should try to use transgenic or gene editing technology to modify the structure or expression of IQD in plants. For example: transfer the osIQD14 gene of rice into wheat or corn to increase their production? Transform the drought resistance genes BrIQD5 in cabbage into wheat and corn to promote insistence level. This could be a direction for future exploration.

## Author Contributions

CG wrote the manuscript and drew the figures. JZ revised the manuscript. DL conceived the study and edited the manuscript. All authors contributed to the article and approved the submitted version.

## Conflict of Interest

The authors declare that the research was conducted in the absence of any commercial or financial relationships that could be construed as a potential conflict of interest.
